# Detection of viruses from feces of wild endangered *Macaca maura*: a potential threat to moor macaque survival and for zoonotic infection

**DOI:** 10.1186/s12917-022-03506-y

**Published:** 2022-11-29

**Authors:** Giusy Cardeti, Antonella Cersini, Giuseppe Manna, Paola De Santis, Maria Teresa Scicluna, Alessandro Albani, Massimiliano Simula, Stefania Sittinieri, Laura De Santis, Claudio De Liberato, Putu Oka Ngakan, Isra Wahid, Monica Carosi

**Affiliations:** 1 Istituto Zooprofilattico Sperimentale del Lazio e della Toscana “M. Aleandri”, Rome, Italy; 2 Department of Sciences, Roma Tre University, Rome, Italy; 3 Royal Society for the Protection of Birds/Gola Rainforest National Park, Kenema, Sierra Leone; 4 Faculty of Forestry, Hasanuddin University, Makassar, Sulawesi Indonesia; 5 Faculty of Medicine, Hasanuddin University, Makassar, Sulawesi Indonesia

**Keywords:** Wildlife, Sulawesi, Non-human primates, *Macaca maura*, Enteric virus, Transmissible, nsEM, PCR

## Abstract

**Background:**

To date, there is a scarcity of information and literature on *Macaca maura* health status relative to viral diseases. The objectives of the present study were to investigate on the potential spread of enteric and non-enteric viruses shed in the environment through a wild macaque feces and to understand the possible interrelation in the spread of zoonotic viruses in a poorly studied geographical area, the Sulawesi Island. This study will also contribute providing useful information on potential threats to the health of this endangered species.

**Methods:**

The sampling was conducted between 2014 and 2016 in the Bantimurung Bulusaraung National Park, in the south of the Sulawesi Island and non-invasive sampling methods were used to collect fresh stools of the *M. maura*, one of the seven macaque species endemic to the island of Sulawesi, Indonesia. The population under study consisted in two wild, neighboring social macaque groups with partially overlapping home ranges; twenty-four samples were collected and examined using negative staining electron microscopy and a panel of PCR protocols for the detection of ten RNA and two DNA viruses.

**Results:**

Viral particles resembling parvovirus (5 samples), picornavirus (13 samples) and calicivirus (13 samples) were detected by electron microscopy whereas the PCR panel was negative for the 12 viruses investigated, except for one sample positive for a mosquito flavivirus. The results did not correlate with animal sex; furthermore, because all of the animals were clinically healthy, it was not possible to correlate feces consistency with viral presence.

**Conclusions:**

As information on viral infections in wild moor macaques remains limited, further studies are yet required to identify the fecal–oral and blood transmitted potentially zoonotic viruses, which may infect the moor macaque and other macaque species endemic to the South Sulawesi Island.

**Supplementary Information:**

The online version contains supplementary material available at 10.1186/s12917-022-03506-y.

## Background

Wild and domestic animals can act as natural reservoirs or amplifying hosts for zoonotic pathogens, in particular viruses [1]. Non-human primates (NHP) may serve as the most suitable model for pathogen transmission to humans, due to their immunological and physiological similarities [2]. On the other hand, endangered species can be at risk of lethal viral infections transmitted by humans [3]. In fact, among Old World monkeys, the macaques are the most studied primates owing to their susceptibility to several human viral infections [4, 5].

According to epidemiological surveys conducted in three national primate research centers and three zoos in the United States [5], captive NHP colonies are seasonally affected by viral infections causing diarrhea [6, 7]. The most studied groups of enteric viruses in NHPs are the monkey rotaviruses and rhesus enteric caliciviruses (ReCV), found in the green vervet monkey (*Chlorocebus pygerythrus*), the Rhesus macaque (*Macaca mulatta*) and the pig-tailed macaque (*Macaca nemestrina*) [8, 9]. However, serological and virological investigations indicate that numerous other NHP species, both in captivity and in the wild, are infected by or become seropositive to these virus families [10, 11].

Other enteric viruses that may induce gastroenteritis in humans and in captive and semi-wild NHP, are represented by DNA viruses such as adenovirus (fam. Adenoviridae), annellovirus (fam. Annelloviridae), and smacovirus (fam. Smacoviridae), and RNA viruses such as enterovirus (fam. Picornaviridae), picobirnavirus (fam. Picobirnaviridae), coronavirus (fam. Coronaviridae), sapovirus (fam. Caliciviridae), astrovirus (fam. Astroviridae) and parvovirus (fam. Parvoviridae) [12–16]. It is important to emphasize that in studies conducted on both ill and healthy animals in zoos, wildlife parks and research centers, many of these viruses are found in both symptomatic (diarrheal) and asymptomatic individuals.

Below we provide a list of other RNA and DNA viruses known to induce similar diseases in man, as well as in NHP (Table 1).

**Table 1:** List of RNA/DNA virus known to induce similar disease in man and NHPs

Virus (family)	Nucleic acid	Route of infection	Infection in NHPs	World diffusion
Encephalomyocarditis virus (EMCV) (Picornaviridae)	RNA	Fecal–oral	Frequently cause fatal outbreaks in several vertebrates, including NHPs of the genus Macaca [17, 18]	Widespread virus isolated from many domestic and wild animal species [19]
Measles virus (Paramyxoviridae)	RNA	Aerosol	Respiratory and gastrointestinal signs in NHPs including macaque species such as *M. mulatta*, *M. fascicularis*, *M. radiata*, and *M. cyclopis* [20]	Serological evidence of measles infection in several populations of free ranging NHPs in Sulawesi (Indonesia) [21]
Yellow fever virus (YFV), dengue virus (DENV), Zika virus (ZIKV), West Nile virus (WNV) and Usutu virus (USUV) (Flaviviridae)	RNA	Mosquito-borne	Encephalitis [22]. WNV isolated in *M. sylvanus* [23]; USUV infect cell lines or primary cells representing different vertebrate species, including monkeys [24]	Usutu virus is restricted to Africa and Europe, where it affects both mammals and birds
Influenza type A viruses (Orthomyxoviridae)	RNA	Aerosol	Replicate in the upper respiratory tract of laboratory macaques, causing either asymptomatic or mild clinical infections [25]	Antibodies to influenza A were detected in sera of both pet and wild Tonkean macaques in Sulawesi (Indonesia) [21]
Hepatovirus A (HAV) (Picornaviridae)	RNA	Fecal–oral	Self-limiting hepatitis A in a variety of primate species, including macaque species such as the rhesus macaque, the cynomolgus macaque, the stump-tailed macaque (*M. arctoides*), and the black macaque (*M. nigra*) [26]	Antibodies to HAV were detected in sera of rhesus macaque in Brazil [27]
Hepatovirus E (HEV) (Hepeviridae)	RNA	Fecal–oral	Causative agent of self-limiting hepatitis E	Antibody responses reported in wild-caught macaque species suggesting natural infections; infectivity studies have demonstrated that the cynomolgus macaque and rhesus macaque are capable of transmitting HEV [28]
Herpesvirus (HV) (Herpesviridae)	DNA	Sexual or biting behavior and by fomites	Zoonotic monkey Herpes B virus causes an extremely serious and usually fatal infection in man [29]	Associated with many important diseases in humans and were reported to occur also in NHPs [26]
Orthopoxvirus (OPV) (Poxviridae)	DNA	Fecal–oral-nasal and skin contact	Fatal infections reported in captive Barbary macaque [30] and in Tonkean macaque [31]	Associated with many important diseases in humans and were reported to occur also in NHPs

**Fig. 1:**
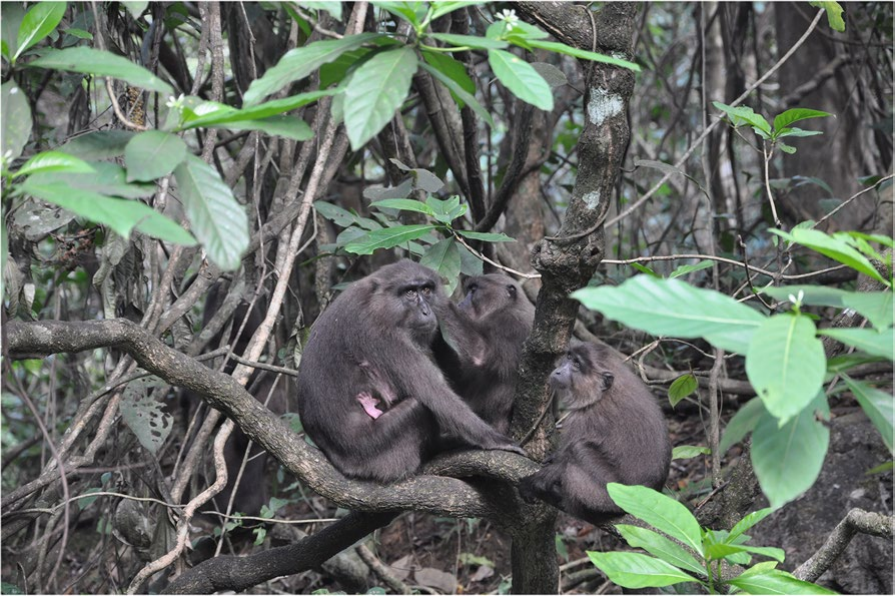
Wild moor macaques (*Macaca maura*) in Karaenta (Bantimurung Bulusaraung National Park, Indonesia) (reproduced with permission from Cristina Sagnotti)

The moor macaque (*Macaca maura*) (Fig. 1) is one of the seven macaque species endemic to Sulawesi, and is listed as Endangered in the International Union for Conservation of Nature (IUCN) Red List of Threatened Species, due to habitat disturbance and fragmentation [32]. As most of Sulawesi Southern district’s lowland tropical rainforest was heavily modified by human encroachment, residual populations of *M. maura* are mostly relegated to a more inaccessible (but not isolated) habitat, the karst forest [33], which likely provides a natural refuge from intrusive and highly impactful human disturbance. Notably, the only other non-human primate species found in this forest is the Makassar tarsier (*Tarsius fuscus*).

The purpose of the investigation on the presence of enteric and non-enteric viruses in the faeces of M. maura was twofold: a) to detect the potential spread of viruses in the environment by infected macaques through the fecal route; b) identify pathogens in feces, potentially harmful to this endangered species and transmitted to it by humans or other animals. Wild macaques could indeed transmit viruses to other animals including humans, but humans or any other mammal residing in the same forest could also infect them. Relative to zoonotic viral infections, the associated risk may depend substantially on three factors: 1) visitors spreading a virus, 2) an NHP comes into close contact with an infected individual (human or NHP) or with contaminated fomites and 3) an NHP is naive to a virus and therefore is highly susceptible to it [20].

## Methods

Our research was conducted exclusively using non-invasive methods; capture or direct contact with the animal were never necessary. For ethics approval and consent to participate, see Declarations section.

### Time frame and site of study

**Fig. 2:**
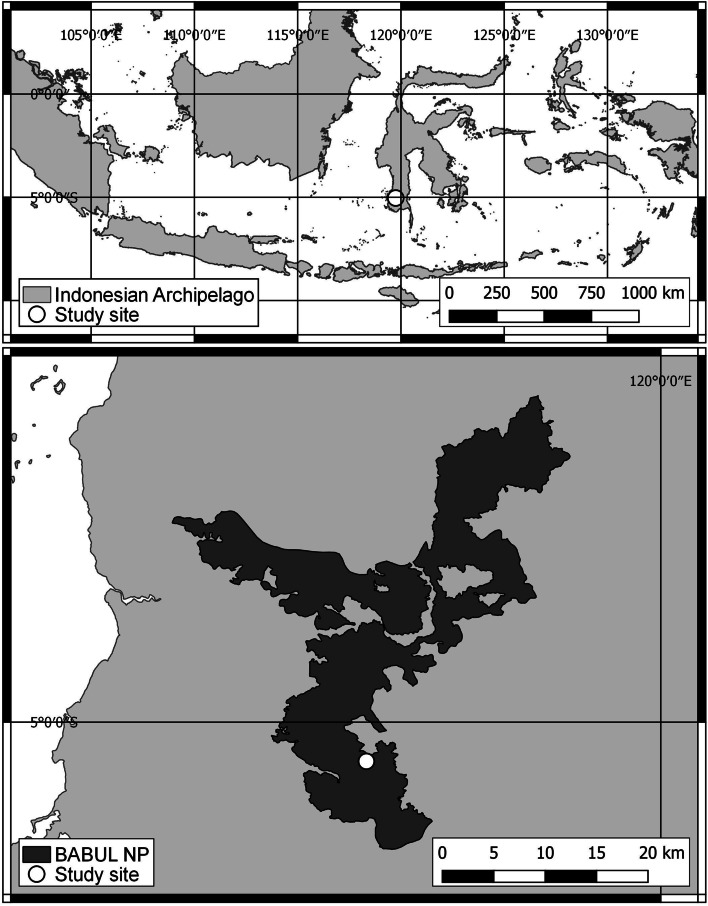
Indonesian archipelago, BABUL NP and the study site on South Sulawesi Island

The study was conducted from 2014 to 2016 in the Karaenta, former Nature Reserve now included in Bantimurung Bulusaraung National Park (hereafter BBNP; 119° 34′ 17′′ - 119° 55′ 13′′ E longitude and 4° 42′ 49′′ - 5° 06′ 42′′ S latitude), South Sulawesi Province, Indonesia (Fig. 2). The study site (258-362 m a.s.l.) is characterized by a karst landscape of tertiary carbonates and represents some of the best-preserved patches of forest of the entire Province. On average, the area receives <60 mm/month and >100 mm/month of rain respectively during the dry and the wet season [34], and the vegetation is typically that of a karst forest [35].

### Epidemiology of the moor macaques

*M. maura* is a social species as almost all primates are, therefore individuals (males and females of different age classes) live organized in stable social groups which represent the functional unit usually studied. Groups are usually named, in order to help primatologists to study the same group over the years and if necessary/possible follow the group members (individually identified) during their lifespan. In the present study, two neighboring social groups, named as Group B [36, 37]  and Group G [38] with partially overlapping home ranges were studied and fecal samples collected. Several behavioral studies were conducted on macaque Group B since 1982 [36, 37], including studies on their reproductive biology, feeding ecology, and habitat use [33, 39, 40]. At the time of our study, Group B comprised 30 ± 1 individuals (4 adult males, 8 adult females and 18±1 immatures). Group G habituation to the human presence started in 2014-2015 [38] and was continued by Carosi and collaborators in 2016. At the time of the study, Group G comprised 18 ± 1 individuals (2 adult males, 4 adult females, and 12 ± 1 immatures). Human exposure was different for the two groups, as Group B was occasionally exposed to human encounters due to a provincial road bisecting its home range, while Group G very rarely interacted with humans.

### Sample collection and identification

Stool samples were obtained non-invasively (i.e., opportunistically when the subjects spontaneously defecated) from individually known subjects during daily behavioral observations and no contact or trapping was needed. Those from Group B were obtained between September 2014 and November 2016, those from Group G in September 2016. The fecal material was collected immediately after being evacuated and involved sampling only the innermost part to avoid potential contamination with soil or other biological agents [41]; the consistency of the sample was determined at the time of collection and classified as formed, soft or loose. Each collection vial containing fecal preservative EcoFix®, (Meridian Bioscience) was filled to the indicated level and shaken to ensure effective storage. All samples fixed with EcoFix® were kept at field temperature for one week before being transported to the laboratory. Fecal samples were collected from known individuals only and first analyzed for helminths and protozoa [42].

The animals were individually identified based on their morphological characteristics and assigned a name. Each sample collected from these individuals was labelled reporting the subject’s name, sex and date of collection. On arrival to the laboratory, the samples were identified with a progressive number for easier reference.

We investigated the presence of enteric and non-enteric viruses in 24 samples of 18 macaques belonging to the two groups as reported in Table 2. Even if some subjects are under-represented due to individual specific behavioral characteristics and/or differences in individual habituation levels, at least one sample per individual was analyzed. For six of the 14 individuals representing group B, another sample is provided (one per survey season). The information on the individuals of the two study groups from which the samples were collected and fecal consistency are reported on Table 3.

**Table 2:** Number of subjects enrolled in the study, group membership, and samples per subject

Social Group membership	No. of subjects	No. of subjects (No. of fecal samples/subject)		Total fecal samples (males + females)
		**Male**	**Female**	
“B”	14	2 (2)	4 (2)	12 (4 + 8)
		2 (1)	6 (1)	8 (2 + 6)
“G”	4	2 (1)	2 (1)	4 (2 + 2)
**Total**	18	6 (8)	12 (16)	24 (8 + 16)

**Table 3:** Macaque group, subject name and sex of individuals from which fecal samples were obtained (including date of collection and consistency), South Sulawesi Province, Sulawesi Island, Indonesia

N	Subject name	Sex	Group	Date of collection	Consistency of feces
1	Jay	Male	B	09/10/2014	Formed
2	Pad	Male	B	28/10/2014	Soft
3	Nop	Female	B	29/10/2014	Formed
4	Eli	Female	B	07/11/2014	Formed
5	Cac	Female	B	28/11/2014	Soft
6	Bet	Female	B	17/12/2014	Soft
7	Hen	Male	B	21/01/2015	Formed
8	Fin	Female	B	26/01/2015	Soft
9	Lan	Female	B	06/02/2015	Soft
10	Pin	Male	B	18/02/2015	Formed
11	Put	Female	B	30/07/2016	Formed
12	Fin	Female	B	02/08/2016	Loose
13	Bet	Female	B	29/08/2016	Formed
14	Af2	Female	G	06/09/2016	Soft
15	Af3	Female	G	15/09/2016	Soft
16	Am2	Male	G	15/09/2016	Formed
17	Am1	Male	G	20/09/2016	Loose
18	Eli	Female	B	20/09/2016	Unavailable
19	Tit	Female	B	20/09/2016	Unavailable
20	Nop	Female	B	04/10/2016	Soft
21	Jay	Male	B	04/10/2016	Unavailable
22	Hen	Male	B	03/11/2016	Unavailable
23	Cac	Female	B	04/11/2016	Soft
24	Lan	Female	B	08/11/2016	Loose

### Method tests used

#### Negative staining electron microscopy

The 24 fecal samples were prepared for negative staining electron microscopy (nsEM) [43] using 2% (w/v) phosphotungstic acid (pH 6.6) and support 400 mesh copper grids, covered with a carbon reinforced plastic film. Each sample (approx. 1g) was ground in 5 ml of sterile distilled water (20% w/v) to form a suspension and clarified by two successive centrifugations at 3000 *g* for 30 min and at 9000 *g* for 30 min. A volume (80 μl) of the supernatant was ultracentrifuged in Airfuge Beckman for 20 min at 21 psi (82000 *g*) and pelleted on a formvar-coated grid. Each grid was subjected before use, to alcian blue staining, to ensure that they were highly hydrophilic and then placed onto a drop of 2% phosphotungstic acid (pH 6.6) for two minutes to counter-stain the grid; excessive stain was removed via three washes in reagent grade water and desiccated by dry adsorption. Analysis of each sample was undertaken using a Philips EM 208 transmission electron microscope (TEM) at 28000 magnifications at 80 kilovolts. For the detection of the virus particles, analysis time (sample grid viewing) was standardized to about 20 minutes per sample.

#### Assessment of the effect of Ecofix® on the detection of viruses using PCR

Viral DNA and RNA was extracted from the macaque feces preserved in EcoFix^®^solution (Meridian Bioscience). The effect of EcoFix^®^ preservative on the detection of viruses by PCR was assessed by adding 10 μl Mengovirus control (strain MC0, 1.6×10^5^ TDCI50/ml) to a 1 ml aliquot of EcoFix^®^ solution to mime field conditions. For this, 5 separate vials were prepared as described above and were kept at 37° C (above normal South Sulawesi field temperature) for one week and subsequently transferred to 5° C for an additional week and finally assayed using real time RT-PCR to establish the percentage of Mengovirus control recovered [44].

#### RNA and DNA viral extraction

According to the extraction protocols recommended for Flavivirus, coronavirus, EMCV, morbillivirus (MV), herpesvirus (HV), WNV, USUV, influenza A virus, orthopoxvirus (OPV), 100 μg of each stool was placed into a two ml plastic vial with a 5 mm stainless steel grinding ball and 1 ml of Buffer, ground with a Tissue Lyser II (QIAGEN, GmbH, Hilden, Germany) at 30 Hz for 3 min, followed by a centrifugation at 17 *g* for 10 min at 4° C. A volume of 200 μl of the supernatant was used for the nucleic acid extraction by the kit QIAamp^®^ cador Pathogen Mini Kit (QIAGEN, GmbH, Hilden, Germany). Five μl of eluate, from a total volume of 60 μl, was used for the PCR protocols that are described further on.

For HAV, HEV and Norovirus the method described by Szabo et al. (2015) [45] with slight modifications, was used. Each Ecofix^®^ preserved sample of macaque feces was homogenized and as described in more detail in an additional file (see Additional file 1) 200 mg were used for viral RNA purification. RNA was resuspended in a final volume of 100 µl of molecular grade water. One ml of molecular grade water was included in each sample extraction batch as negative extraction control.

The concentration and purity of the extracted nucleic acids was evaluated using spectrophotometric analysis based on the absorbance values (A) at the wavelengths of 260 and 280 nm.

#### PCR protocols for RNA and DNA virus detection

The PCR protocols used for RNA and DNA virus detection are listed in Table 4.

**Table 4:** PCR Protocols for RNA and DNA virus detection

RNA Virus	PCR Protocol	References
Flavivirus	cDNA synthesis followed by PCR end point	[46], Additional file 1
Coronavirus	cDNA synthesis followed by PCR end point	[47], Additional file 1
EMCV	cDNA synthesis followed by PCR end point	[48], Additional file 1
EMCV	Real time RT-PCR	Kindly provided by dr E.A. Foglia, IZS Lombardia ed Emilia Romagna, Italy
Morbillivirus	cDNA synthesis followed by PCR end point	[49], Additional file 1
WNV Lineage 1 and 2	Real time RT-PCR	[50], Additional file 1
USUV	Real time RT-PCR	[51], Additional file 1
Influenza A virus	Real time RT-PCR	[52], Additional file 1
HAV	Real time RT-PCR	[53], Additional file 1
HEV	Real time RT-PCR	[54, 55], Additional file 1
Norovirus genogroup I (GI) and II (GII)	Real time RT-PCR	[53–55], Additional file 1
**DNA Virus**	**PCR Protocol**	**References**
Herpesvirus	nested PCR	[56], Additional file 1
Orthopoxvirus (OPV)	SYBR Green real time PCR	[57], Additional file 1
Parvovirus	Quanty® Parvo B19 kit	Additional file 1

## Results

Twenty-four samples were collected from 18 subjects for which six animals had two samples, collected at different times, as described in Table 2.

### Negative staining electron microscopy

Electron microscopy of negative-stained suspensions prepared from feces of moor macaques detected different viral particles as reported in Table 5.

**Table 5:** Results of virological investigations on fecal specimens from wild moor macaques

**Sample** **N**	**nsEM**	**RNA Virus – PCR (EMCV, MV, Coronavirus, Flavivirus**^a^**, WNV, USUV, Influenza A, HAV, HEV, Norovirus)**	**DNA Virus – PCR (HV, OPV)**
1	Phages; particles of 70 nm in diameter	Negative	Negative
2	Phages; Parvolike; Picornalike; 2 bacteria; bacterial pili; 70 nm particles	Positive only for *Culex flavivirus* Sequence ID: KF022194.1 Id. 100%	Negative^b^
3	Phages; Picornalike	Negative	Negative
4	Negative	Negative	Negative
5	Phages; 70 nm particles	Negative	Negative
6	Picornalike; 70 nm particles	Negative	Negative
7	Picornalike; bacterial pili; 70 nm particles	Negative	Negative
8	Phages; Picornalike; bacterial pili; 55 nm and 70 nm particles	Negative	Negative
9	Icoshaedral particles of 70 nm in diameter	Negative	Negative
10	Bacterial pili	Negative	Negative
11	Phages; Picornalike; bacterial pili; 70 nm particles	Negative	Negative
12	Phages; Parvolike; Picornalike; 70 nm particles	Negative	Negative^b^
13	Phages; Picornalike; 70 nm particles	Negative	Negative
14	Phages; Calicilike; turret shape particles of 50 nm in diameter; 70 nm particles	Negative	Negative
15	Phages; Calicilike; Parvolike; turret shape particles of 50 nm in diameter	Negative	Negative^b^
16	Picornalike; empty turret shape particles of 70 nm; 50 and 120 nm particles	Negative	Negative
17	Phages; Picornalike; empty 50 nm particles; plant structures	Negative	Negative
18	Picornalike; turret shape 50 nm particles	Negative	Negative
19	Picornalike; bacterial pili; 70 nm particles; empty 50 nm particles	Negative	Negative
20	Particles of 50 nm; several particles of 115–120 nm in diameter	Negative^c^	Negative
21	Parvolike; empty and full particle of 50 nm	Negative	Negative^b^
22	Parvolike; bacterial pili; particles of 70 and 120 nm; plant structures	Negative	Negative^b^
23	Phages; Picornalike; particles of 70 and 120 nm in diameter	Negative	Negative
24	Picornalike; empty particles of 70 nm; 50 nm particles; plant structures	Negative	Negative

^a^Dengue 1, 2, 3 and 4; YFV, WNV, JEV, MVE, SLE and USUV

^b^five samples analyzed with Quanti® Parvo B19 kit (Clonit)

^c^the sample was analyzed with two EMCV PCR protocols

**Fig. 3:**
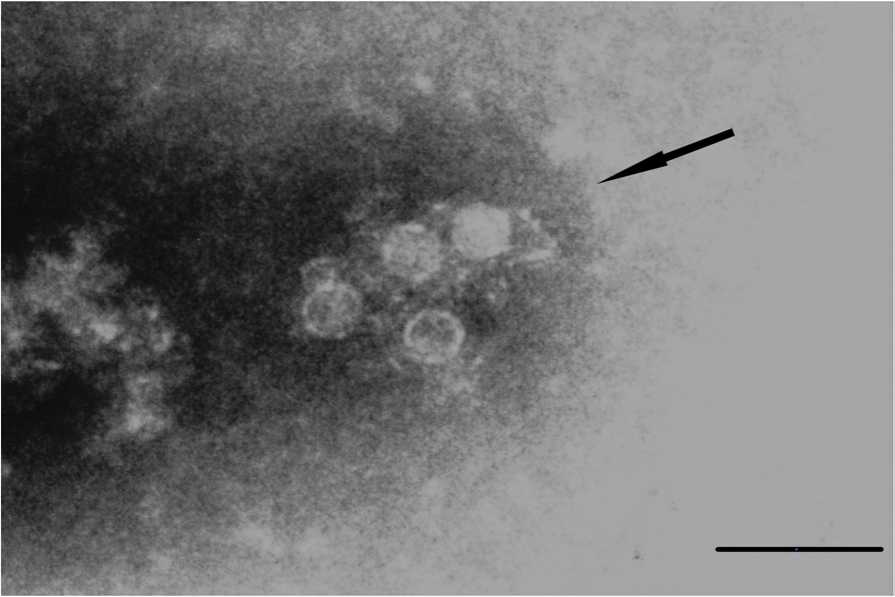
Electron micrograph. Picornalike-virus particles (arrow) at nsEM (2% PTA. Bar = 100 nm)

Small, non enveloped icosahedral viral particles, approximately 18-22 nm in diameter, morphologically resembling parvovirus were found in five samples and icosahedral viral particles, approximately 25-30 nm in diameter, morphologically resembling virus from *Picornaviridae* family in 13 samples (Fig. 3); calicivirus-like particles were found in two samples while bacteriophages were detected in 13 samples (two of them belonging to the same individual). No correlation was found between nsEM results and animal sex, stool consistency and PCR results.

### DNA and RNA purity and PCR analyses

The A260/280 nm ratios of the extracted DNA and RNA were 1.8/2 respectively, and both considered acceptable for purpose of use.

For HAV, HEV, Norovirus GI and GII real time RT-PCR, the use of an EC for each target in the samples analyzed, allowed PCR inhibition assessment and was found to conform to the accepted value of ≤ 50%.

For all other real time PCR assays, an exogenous internal positive control (IPC), (Kit TaqMan Exogenous Internal Positive Control, Cat. 4308323, Applied Biosystems) was used. All tested stool samples showed IPC amplification with values ranging from Ct 28 to 30, that is considered as acceptable.

The viral extraction efficiency, calculated as percentage of process control Mengovirus recovery, also conformed to the accepted value of ≥ 1% for all samples processed for HAV, HEV, Norovirus GI and GII.

Similar values were obtained when the five vials of Ecofix^®^ with Mengovirus were assayed using real time RT-PCR (range 1.7-4.6 %).

The results of the various PCR protocols are described in Table 5.

## Discussion

As part of a long-term project on the behavioral ecology and reproductive biology of the Moor macaque [33] and add to the knowledge of its gastro-intestinal parasitic infections [42], we investigated possible viral infections in wild moor macaques and the consequent potential to contaminate the environment through infected feces. In addition, we attempt to assess the risk of infection for the moor macaque population and other cohabiting animals and humans that share the same territory. Another factor to consider is the continuous human population growth in the area, small-scale forest use (e.g. harvesting of forest products such as honey and palm fermented sap) still occurs in the National Park for subsistence, and for this reason it is possible for *M. maura* to come into contact with humans in their natural habitat [58].

The inability to molecularly detect nucleic acid is not attributed to the use of the EcoFix^®^ fixative, as other authors were able to detect viruses in human feces conserved in this reagent [59]. Furthermore, in our study, both the values recorded by spectrophotometry for DNA and RNA and the IPC amplifications were considered to be of good quality for PCR analysis, and the morphology of the viral particles in the samples observed in the nsEM were conserved.

As it was not possible to exclude that the viral particles observed at TEM were unknown, poorly known or plant-derived species, additional experiments should be conducted in future to either detect or exclude the presence of viral pathogens, especially in view of the EM results obtained. In this regard, metagenomic studies could be designed to fish out viral nucleic acids of potential zoonotic interest.

Some picornavirus-like particles were observed in nsEM, but they were not identified as EMCV or Hepatitis virus A and E using targeted PCR protocols.

The only flavivirus detected by PCR is one associated with mosquitoes of the genus *Culex*, possibly attributable to an infected insect contaminating the fecal sample before collection.

All the wild moor macaques analyzed were norovirus negative, leading us to hypothesize that at the time of sampling the virus was not circulating in their habitat. Rotavirus and norovirus are known to occur in in many domestic and wild animal species worldwide [60, 61] and they have been reported in the human population of Indonesia as well [62]. Nowadays, as humans are increasingly interacting with at least one of the macaque social groups we investigated [63], a new potential risk for macaque might be hypothesized. Fortunately, as far as we know, there is no published information available on the occurrence and spread of norovirus in the human and animal populations of Sulawesi to date.

The observed presence of some calicivirus-like particles under nsEM was not confirmed by the PCR results.

Parvo-like particles were observed in five feces samples using nsEM. These samples were molecularly assayed using the commercial kit Quanty^®^ Parvo B19 (Clonit), specifically designed to detect Parvovirus B19. Parvovirus similar to B19 are known to infect Simian erythrocytes; accordingly, the virus particles observed in feces using nsEM could be referred to as parvo-like viruses.

Several particles representing phages were observed in the samples assayed. While in domestic animals it often means an imbalance in the intestinal flora, in wild moor macaques, phage presence may be linked to a natural diet and different environment [64].

The limited knowledge currently available on the viruses that infect wild moor macaques, the limitations of electron microscopy, combined with the targeted and narrowed specificity of particular PCR protocols, together may explain the lack of identified pathogenic and non-pathogenic viruses. Furthermore, all NHP subjects appeared to be in good health during the prolonged period of observation and never displayed any signs that could be associated with a clinical infection.

## Conclusions

In this study, we investigated the presence of viruses excreted in feces by macaques, and which may infect other animals of the same group or other macaque and NHP species. All the individuals included in the study, and living in a protected area, were apparently in good health; some of them excreted soft or loose feces, but it was not possible to associate this state with a viral infection. In this study, all moor macaque feces tested negative for the viruses investigated, but the fact that at the time of the study there was no active elimination of the viruses investigated, does not exclude possible latent viral infections e.g. herpesvirus. Indeed, expanding on the study population and area and extending the period of sampling would increase the evidence on the effective virus circulation not only for the southern part of the Sulawesi Island.

The program for the maintenance of the specific pathogen free state of captive NHPs is focused on blood-transmitted pathogens such as simian immunodeficiency virus (SIV); simian T lymphotropic virus type 1 (STLV); simian retrovirus type D (SRV), and herpes B virus. However, given the significant morbidity associated with viral diarrhea, preventive measures in these animals should also address enteric pathogenic viruses that are endemic, both among macaque colonies and in the surrounding environment [6, 12]. On the other hand, further studies are required to highlight the possible circulation of viruses transmitted by both blood and feces that could infect not only the moor macaque but also other macaque species endemic in Sulawesi and South-East Asia. To date, the Global Mammal Parasite Database catalogues only two studies on deltaretrovirus infection in wild moor macaque [65].

Of potential research interest would be to serologically and virologically analyze saliva found on ropes laced with appetent on which the animals can chew. This represents another non-invasive method for sample collection, useful to investigate the health history and to detect other pathogens that may circulate within this endangered NHP species [65–67].

## Supplementary Information

**Additional file 1.**

## Data Availability

All data generated and/or analyzed during this study are included in this published article [and its supplementary information files].

## Abbreviations

BBNP: Bantimurung Bulusaraung National Park
DENV: Dengue virus
DNA: Deoxyribonucleic acid
EC: External amplification control
EMCV: Encephalomyocarditis virus
GI: Genogroup I
GII: Genogroup II
HAV: Hepatovirus A
HEV: Hepatitis E
HV: Herpesvirus
IPC: Internal Positive Control
ISO: International Organization for Standardization
IUCN: International Union for Conservation of Nature
MV: Morbillivirus
NHP: Non-Human Primate(s)
nsEM: Negative staining electron microscopy
OPV: Orthopoxvirus
PVB19: Parvovirus B19
ReCV: Rhesus enteric Calicivirus(s)
RNA: Ribonucleic acid
RT-PCR: Reverse Transcriptase-Polymerase Chain Reaction
TEM: Transmission Electron Microscope
USUV: Usutu virus
WNV: West Nile virus
YFV: Yellow fever virus
ZIKV: Zika virus
