# Supplementary material for: Detection of viruses from feces of wild endangered Macaca maura: a potential threat to moor macaque survival and for zoonotic infection
Source: BMC Vet Res. 2022 Nov 29;18:418. doi: 10.1186/s12917-022-03506-y (PMC9706849; doi:10.1186/s12917-022-03506-y)
Supplement: Supplementary file 1 — Additional file 1. [file 12917_2022_3506_MOESM1_ESM.docx]

**PCR PROTOCOLS**

**PCR PROTOCOLS FOR RNA DETECTION**

1. Flavivirus

Panflavivirus PCR can detect the following viruses: Dengue 1 and 2 (854bp), Dengue 3 and 4 (285bp), Yellow Fever virus (YFV), West Nile virus (WNV), Japanese Encephalitis virus (JEV), Murray Valley Encephalitis virus (MVE), St. Louis Encephalitis virus (SLE) and USUTU group (863bp) [1]. The cDNA synthesis was performed using the High Capacity cDNA Reverse Transcription kit (Applied Biosystems, ThermoFisher Scientific). Briefly, 30 µl of purified RNA, 6 µl of 10X random hexamer primers, 6 µl 10X RT-Buffer, 2.4 µl dNTP mix 100mM, 3 µl of 5U Multi Scribe Reverse Transcriptase and 12.6 µl of RNase-free water were added to each sample.

The PCR was performed using the Platinum®Taq DNA Polymerase kit (Invitrogen, ThermoFisher Scientific) and carried out in 50 μl final volume with 5 μl of Buffer 10X, 2 μl (2mM) of MgCl2 50mM, 1 μl (0.2μM) of dNTPs 10mM, 1 μl (0.6μM) of each primer FLAVI-1 and FLAVI-2, 5 μl of cDNA template and 0.05 U/μl of Platinum Taq DNA Polymerase 5 U/µl. The PCR was performed using the GeneAmp PCR System 9700 (Applied Biosystems, Foster City, CA, USA)

1. Coronavirus [2]

Pancoronavirus PCR required cDNA synthesis as previously described for Panflavivirus PCR using AmpliTaq Gold® DNA Polymerase (Applied Biosystems, Life Technologies, Austin, Tx, USA). It was carried out with 5 μl of Buffer 10X, 6 μl (3mM) of MgCl2 25mM, 1 μl (0.2μM) of dNTPs 10mM, 1 μl (0.83μM) of each primer COR-F and COR-R, 5 μl of cDNA template and 0.07 U/μl of AmpliTaq DNA Polymerase and brought to a 50 μl final volume using DEPC water as for all the protocols described further on. The PCR was performed using the GeneAmp PCR System 9700 (Applied Biosystems, Foster City, CA, USA)

1. Encephalomyocarditis virus [3]

EMCV PCR requires cDNA synthesis as previously described and the PCR was performed using High Fidelity Platinum®Taq DNA Polymerase (Invitrogen, ThermoFisher Scientific) and carried out in 50 μl final volume with 5 μl of Buffer 10X High Fidelity, 2 μl (2mM) of MgSO4 50mM, 1 μl (0.2mM) of dNTPs 10mM, 1 μl (1μM) of each primer 3DP1 and 3DP2, 5 μl of cDNA template and 0.05 U/μl of High-Fidelity Platinum®Taq DNA Polymerase. The EMCV PCR amplifies fragments between 200 and 300bp depending on the strain. The PCR was performed using the GeneAmp PCR System 9700 (Applied Biosystems, Foster City, CA, USA)

1. Morbillivirus [4]

Morbillivirus PCR requires cDNA synthesis as previously described and the PCR amplifies a 287bp fragment located on the conserved N terminus of the morbillivirus NP gene using Platinum® Taq DNA Polymerase (Invitrogen, ThermoFisher Scientific) and carried out in 50 μl final volume with 5 µl of Platinum Taq Buffer 10X, 0.33 µl (0.2µM) of each primer MvF and MvR, 2.5 µl (2.5mM) of MgCl2 50mM, 2 µl (0.4mM) of dNTPs 10mM, 5 µl of cDNA template and 0.5 µl Platinum™Taq DNA Polymerase 5 U/µl (Invitrogen™, ThermoFisher Scientific) at 0.05 U/µl final concentration. The PCR was performed using the GeneAmp PCR System 9700 (Applied Biosystems, Foster City, CA, USA)

All products of the Panflavivirus, Pancoronavirus, EMCV and Morbillivirus PCRs were visualized in electrophoresis using a 1.5% Tris-Boric Acid-EDTA agarose gel and stained with GelRed 10,000X (Biotium, Hayward, CA, USA). Sequencing was performed in case of the presence of an amplicon of the expected size.

For sequencing the PCR product for EMCV and flavivirus have been recovered by QIAquick® PCR Purification kit (QIAGEN, GmbH, Hilden, Germany) according to the manufacturer’s instructions and sequenced using the specific PCR primers with the BigDye Terminator Cycle Sequencing Ready Reaction kit, version 3.1 (Applied Biosystems, Foster City, CA, USA) in an automated sequencer (3500 Genetic Analyzer, A. Biosystems, Foster City, CA, USA). The nucleotide sequences obtained were analysed using the Genetic Analyzer Sequencing v5.4 (A. Biosystems, Foster City, CA, USA).

1. WNV Lineage 1 and Lineage 2, USUV, Influenza A virus

The samples were analyzed by real time reverse transcription (RT)-PCR for WNV Lineage 1 and Lin-eage 2 [5], by real time RT-PCR for USUV [6] and real time RT-PCR for Influenza A virus [7]. The amplification mix used was the AgPath-ID One-Step RT-PCR Reagents. The real time RT-PCR were performed using the Quant Studio 7 Flex System (Applied Biosystems, Foster City, CA, U.S.A.).

1. Hepatitis A virus, hepatitis E virus, norovirus GI and GII viruses [8-12]

An aliquote of 200 mg was used used for viral RNA purification from feces samples was added to 50 ml falcon tubes, containing approximately 20 sterile glass beads (2-4 mm in diameter); subsequently, 10 µl of process control virus (Mengovirus, strain MC0, 1.6×10^5^ TDCI50/ml) and 3.5 ml of QIAzol® Lysis Reagent (QIAGEN, GmbH, Hilden, Germany) were added to each sample and high-speed vortexed for 1 min. After an incubation for 15 min at room temperature, all tubes were centrifuged at 10,000 × g for 20 min at 4 °C and the supernatants transferred to a fresh 15 ml falcon tubes. Chloroform, in the volume of 0.7 ml (0.2 v/v), was added to each tube, and the suspension vortexed for 15 sec. Following further 15 min incubation at room temperature, each sample was centrifuged again at 10,000 × g for 15 min at 4 °C and the recovered supernatant was measured (average 2.2 ± 0.2 ml), recorded and then stored at below -75 °C until processed for nucleic acid extraction. RNA was extracted from 1 ml of sample using the NucliSens MiniMag extraction system (bioMérieux, France) following the manufacturer's instructions. RNA was resuspended.

RNAs obtained from fecal samples were analysed using a 5 µl aliquot as template and using RNA UltraSense™ One-Step qRT-PCR System (Life Technologies) reagents. Analyses were performed in duplicate on a Stratagene MX 3005P (Agilent Technologies, Milan, Italy) thermalcycler. Two no-template negative PCR controls were included during each run. PCR inhibition was ruled out using an external amplification control (EC) (in vitro synthesized RNA) obtained from the National Reference Laboratory for Foodborne Viruses (Istituto Superiore di Sanità, Rome, Italy) and amplification data were considered valid if sample inhibition was ≤ 50%. Viral extraction efficiency from samples was assessed using the process control Mengovirus [13] and set at the efficiency criterion of ≥ 1%; all samples that did not reach ≥1% criterion underwent a second extraction process.

**PCR PROTOCOLS FOR DNA DETECTION**

1. Herpesvirus

Nested PCR (Consensus primer PCR) for the detection of herpesvirus amplifies a conserved region of 215bp-315bp of the DNA polymerase gene [14]. The master mix for PCR1 and PCR2 was performed using AmpliTaq Gold® DNA Polymerase (Applied Biosystems, Life Technologies, Austin, Tx, USA) and carried out in 50 μl final volume with 1μM of each primer, 0.05% DMSO, 2mM MgCl2, 0.2mM dNTPs, 5 μl of template, 5 μl of Buffer 10X and 0.07 U/μl of AmpliTaq DNA Polymerase. The PCR was performed using the GeneAmp PCR System 9700 (Applied Biosystems, Foster City, CA, USA)

1. Orthopoxvirus [15]

SYBR Green real time PCR was performed in a Rotor-Gene instrument (QIAGEN, GmbH, Hilden, Germany), using the QuantiFast SYBR Green PCR Kit (QIAGEN, GmbH, Hilden, Germany).

1. Parvovirus

Nucleic acid was extracted from *Macaca maura* stool samples with Qiasymphony using the DSP Virus/Pathogen Mini Kit (QIAGEN, GmbH, Hilden, Germany). The five nucleic extracts were tested with the commercial Quanty^®^ Parvo B19 kit (Clonit, Italy) which amplifies the VP1 region of the Parvovirus B19 genome.

References

1. Ayers M, Adachi D, Johnson G, Andonova M, Drebot M, Tellier R. A single tube RT-PCR assay for the detection of mosquito-borne flaviviruses. J. Virol. Methods. 2006;135:235-239. doi:10.1016/j.jviromet.2006.03.009.
2. Vijgen L, Moës E, Keyaerts E, Li S, Van Ranst M. A Pancoronavirus RT-PCR Assay for Detection of All Know Coronaviruses. Methods Mol. Biol. 2008;454:3-12. doi:10.1007/978-1-59745-181-9_1.
3. Bakkali Kassimi L, Gonzague M, Boutrouille A, Cruciere C. Detection of Encephalomyocarditis virus in clinical samples by immunomagnetic separation and one-step RT-PCR. J. Virol Methods. 2002;101:197-206. doi:10.1016/s0166-0934(01)00439-6.
4. Verna F, Giorda F, Miceli I, Rizzo G, Pautasso A, Romano A, Iulini B, Pintore MD, Mignone W, Grattarola C, Bozzetta E, Varello K, Dondo A, Casalone C, Goria M. Detection of morbillivirus infection by RT-PCR RFL analysis in cetaceans and carnivores. J. Virol. Methods. 2017;247:22-27.
5. Del Amo J, Sotelo E, Fernández-Pinero J, Gallardo C, Llorente F, Agüero M, Jiménez-Clavero MA. A novel quantitative multiplex real-time RT-PCR for the simultaneous detection and differentiation of West Nile virus lineages 1 and 2, and of Usutu virus. J. Virol. Methods. 2013;189(2):321-7. doi:10.1016/j.jviromet.2013.02.019.
6. Cavrini F, Della Pepa ME, Gaibani P, Pierro AM, Rossini G, Landini MP, Sambri V. A rapid and specific real-time RT-PCR assay to identify Usutu virus in human plasma, serum, and cerebrospinal fluid. J. Clin. Virol. 2011;50(3):221-3. doi:10.1016/j.jcv.2010.11.008.
7. Spackman E, Senne DA, Myers TJ, Bulaga LL, Garber LP, Perdue ML, Lohman K, Daum LT, Suarez DL. Development of a real-time reverse transcriptase PCR assay for type A influenza virus and the avian H5 and H7 hemagglutinin subtypes. J. Clin. Microbiol. 2002;40(9):3256-60. doi:10.1128/JCM.40.9.3256-3260.2002.
8. Svraka S Duizer E, Vennema H, de Bruin E, van der Veer B, Dorresteijn B, Koopmans M. Etiological role viruses in outbreaks of acute gastroenteritis in The Netherlands from 1994 through 2005. J. Clin. Mirobiol. 2007;45:1389-1394. doi:10.1128/JCM.02305-06.
9. da Silva S, Le Saux JC, Parnaudeau S, Pommepuy M, Elimelech M, Le Guyader FS. Evaluation of removal of noroviruses during wastewater treatment, using real-time reverse transcription-PCR: different behaviors of genogroups I and II. Appl. Environ. Microbiol. 2007;73:7891-7897. doi:10.1128/AEM.01428-07.
10. Loisy F, Atmar RL, Guillon P, Le Cann P, Pommepuy M, Le Guyader FS. Real-time RT-PCR for norovirus screening in shellfish. J. Virol. Methods. 2005;123:1-7.
11. Kageyama T, Kojima S, Shinokara M, Uchida K, Fukushi S, Hoshino FB, Tazeda N, Katayama K. Broadly reactive and higly sensitive assay for Norwalk-like viruses based on real-time quantitative reverse transcription-PCR. J. Clin. Microbiol. 2003;41:1548-1557.
12. Le Guyader FS, Parnaudeau S, Schaeffer J, Bosch A, Loysi F, Pommepuy M, Atmar RL. Detection and quantification of norovirus in shellfish. Appl. Environ. Microbiol. 2009;75:618-624.
13. Costafreda MI, Bosch A, Pinto RM. Development, evaluation, and standardization of a real-time TaqMan reverse transcription-PCR assay for quantification of hepatitis A virus in clinical and shellfish samples. Appl. Environ. Micro-biol. 2006;72:3846-3855. doi:10.1128/AEM.02660-05.
14. VanDevanter DR, Warrener P, Bennet L, Schultz E, Coulter S, Garber RL, Rose TM. Detection and Analysis of Diverse Herpesviral Species by Consensus Primer PCR. J. Clin. Microbiol. 1996;34 (7):1666-1671.
15. Carletti F, Di Caro A, Calcaterra S, Grolla A, Czub M, Ippolito G, Capobianchi MR, Horejsh D. Rapid, differential diagnosis of orthopox- and herpesviruses based upon real-time PCR product melting temperature and restriction enzyme analysis of amplicons. J. Virol. Methods. 2005;129:97–100. doi:10.1016/j.jviromet.2005.05.020.
